# Pathological Museum at the Pennsylvania Hospital

**Published:** 1861-01

**Authors:** 


PATHOLOGICAL MUSEUM AT THE PENNSYLVANIA HOSPITAL.—The Board of Managers of this institution recently adopted a resolution appropriating a proper apartment for a Pathological Museum, and Dr. T. G. Morton has been appointed Curator.

